# Recent Advances in Pharmaceutical Cocrystals: From Bench to Market

**DOI:** 10.3389/fphar.2021.780582

**Published:** 2021-11-11

**Authors:** Ravi Kumar Bandaru, Smruti Rekha Rout, Gowtham Kenguva, Bapi Gorain, Nabil A. Alhakamy, Prashant Kesharwani, Rambabu Dandela

**Affiliations:** ^1^ Department of Industrial and Engineering Chemistry, Institute of Chemical Technology-Indian Oil Bhubaneswar Campus, Bhubaneswar, India; ^2^ School of Pharmacy, Faculty of Health and Medical Sciences, Taylor’s University, Subang Jaya, Malaysia; ^3^ Department of Pharmaceutics, Faculty of Pharmacy, King Abdulaziz University, Jeddah, Saudi Arabia; ^4^ Center of Excellence for Drug Research & Pharmaceutical Industries, King Abdulaziz University, Jeddah, Saudi Arabia; ^5^ Department of Pharmaceutics, School of Pharmaceutical Education and Research, Jamia Hamdard, New Delhi, India

**Keywords:** solid dosage forms, pharmaceutical cocrystals, solubility, permeability, cocrystallization, formulation, regulatory guidelines, particle size

## Abstract

The pharmacokinetics profile of active pharmaceutical ingredients (APIs) in the solid pharmaceutical dosage forms is largely dependent on the solid-state characteristics of the chemicals to understand the physicochemical properties by particle size, size distribution, surface area, solubility, stability, porosity, thermal properties, etc. The formation of salts, solvates, and polymorphs are the conventional strategies for altering the solid characteristics of pharmaceutical compounds, but they have their own limitations. Cocrystallization approach was established as an alternative method for tuning the solubility, permeability, and processability of APIs by introducing another compatible molecule/s into the crystal structure without affecting its therapeutic efficacy to successfully develop the formulation with the desired pharmacokinetic profile. In the present review, we have grossly focused on cocrystallization, particularly at different stages of development, from design to production. Furthermore, we have also discussed regulatory guidelines for pharmaceutical industries and challenges associated with the design, development and production of pharmaceutical cocrystals with commercially available cocrystal-based products.

## Introduction

More than 80% of marketed formulations are solid dosage forms intended for oral administration because of their simplicity and high compliance among patients. Since the last decade, efforts have been made to identify the purity and associated side effects of their use. Therefore, the safety and efficacy of the administered therapeutic agents need to be assured. With regard to this concern, the physical properties of the solid dosage forms are a major alarm (Gupta et al., 2015). Therefore, it is of utmost importance to have appropriate solubility and permeability of an active pharmaceutical ingredient (API) to be developed for the oral dosage form, which in turn leads to providing the desired bioavailability in the physiological system to exert its pharmacological role. Along with the abovementioned clinically relevant properties, an API should also possess satisfactory stability, dissolution rate, hygroscopicity, and melting point (physicochemical properties) for better processability during manufacturing. Solid dosage forms, particularly tablets, are the predominant forms for 80% of the commercially available drugs in the market. Among these, 40% of the drugs and a similar percentage of new chemical entities encounter low aqueous solubility (Babu and Nangia, 2011; Gorain et al., 2013). According to the biopharmaceutical classification system (BCS), drug molecules possessing solubility issues are classified under Class II and Class IV (Babu and Nangia, 2011). The origin of this low solubility issue of drugs is a high-throughput screening and combinatorial chemistry program that screened and discovered many drug molecules which are highly lipophilic. For these lipophilic molecules to develop into pharmaceutical products, the solubility profile has to be improved without altering the chemical identity and pharmacological role of the molecule (Thakuria et al., 2013).

**GRAPHICAL ABSTRACT:**
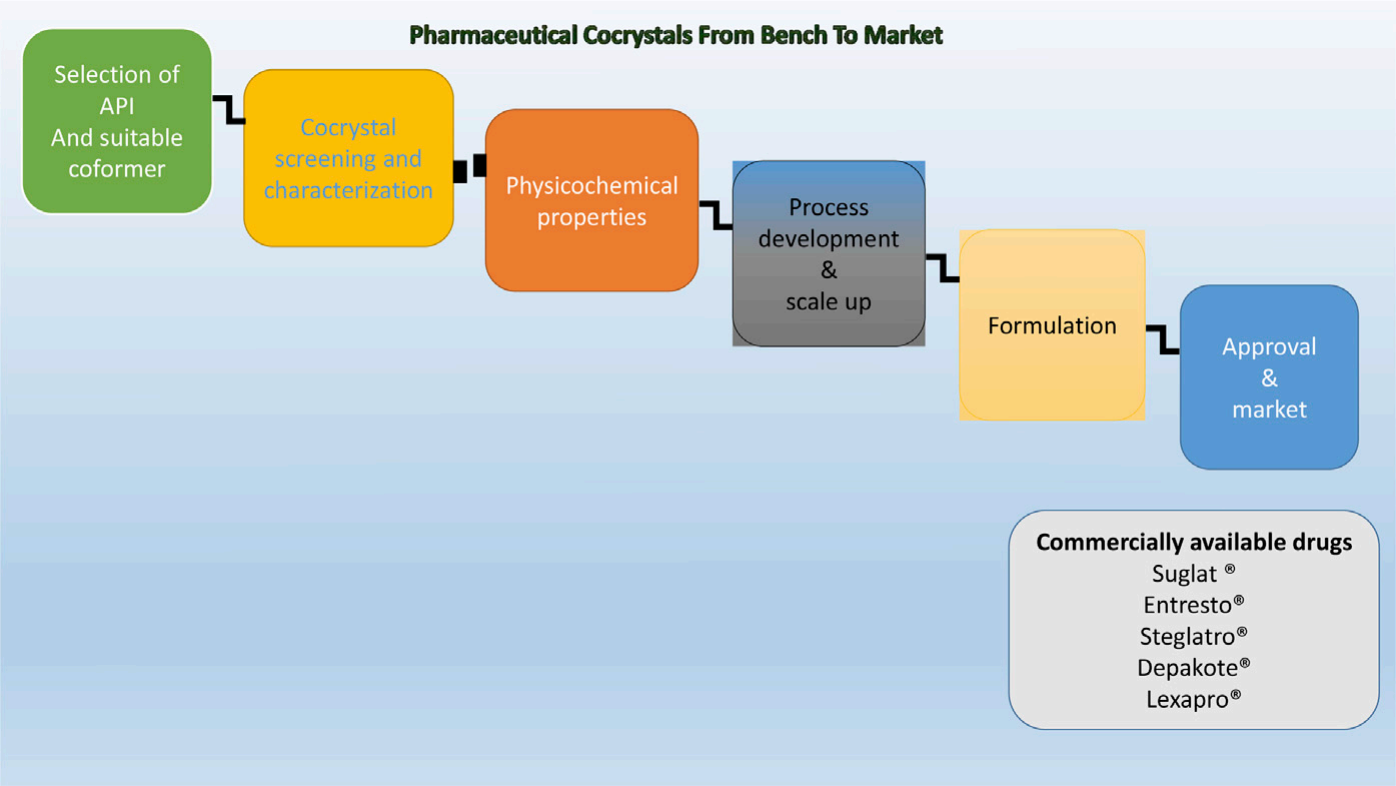


Conventional methods to improve the solubility and other physicochemical properties of APIs include salt formation, polymorphs, solvate or hydrate formation, amorphous forms, etc., and each of these methods has its own limitations. Salt formation is the most widely used technique to improve solubility, which requires sufficient ionizable groups or acidic or basic groups in the drug molecule (Joshi and Choudhury, 2018). Thus, the neutral molecules cannot form salts. Polymorphs, solvates, and hydrates have limited existence. During the last decade, the cocrystallization method was applied to APIs to improve their physicochemical and processability properties without affecting their therapeutic role as a drug (Schultheiss and Newman, 2009; Mannava et al., 2020).

Since solid-state properties are determined by the crystal structure of the corresponding solid, changing the crystal structure by introducing other molecules into the crystal lattice leads to a change in the solid-state properties of the parent solid without altering the actual chemical identity of the native molecule. In this context, cocrystallization is an approach to alter the solid-state properties of a crystalline solid by introducing additional components into the crystal lattice through noncovalent interactions (Friščić and Jones, 2010). According to the United States Food and Drug Administration (USFDA), pharmaceutical cocrystals are crystalline solids having two or more components in their crystal lattice. Thus, solvates and hydrates can also be considered cocrystals. Therefore, the most agreed definition for pharmaceutical cocrystals is crystalline solid forms composed of two or more components in the same crystal lattice, generally in a stoichiometric ratio (Kuminek et al., 2016; Center of Drug Evaluation and Research, 2018), where the components should be solids at room temperature in their pure form (Aitipamula et al., 2012). One of the components of the cocrystal structure is the API molecule, while the other component is called a cocrystal former or conformer. These conformers are generally selected from a list of nontoxic components which are generally recognized as safe (GRAS). Recently, USFDA issued guidelines to pharmaceutical industries regarding pharmaceutical cocrystals. This indicates the increasing prominence of cocrystallization techniques in the pharmaceutical industry. During the last decade, a number of reviews were published discussing the ability of pharmaceutical cocrystals as a way to change the physicochemical properties of APIs (Kuminek et al., 2016). The success of pharmaceutical cocrystals can be attributed to two aspects: 1) scope for design—due to a wide variety of conformers and 2) modularity—the ability of pharmaceutical cocrystals to alter the solid-state properties without affecting their therapeutic activity (Shan and Zaworotko, 2008; Friščić and Jones, 2010) due to noncovalent interactions between component molecules in the cocrystals. These noncovalent interactions include hydrogen bonding, Van der Waal's attraction forces, π-π interactions, and halogen bonding (Stoler and Warner, 2015).

The increasing popularity of this field encourages us to bring a summarized article on cocrystallization technology. Thus, the focus of this review is to discuss cocrystallization techniques adopted by the pharmaceutical industries in designing, developing, and manufacturing suitable solid forms of therapeutics, which have improved clinically relevant physicochemical and processability properties. Furthermore, the review also discusses regulatory guidelines that are currently in force for pharmaceutical cocrystals and their scale-up process. In addition, we have also discussed challenges that are associated with the development of pharmaceutical cocrystals and have listed the currently available cocrystal-based drugs that are available in the market.

## Cocrystallization Techniques

**FIGURE 1:**
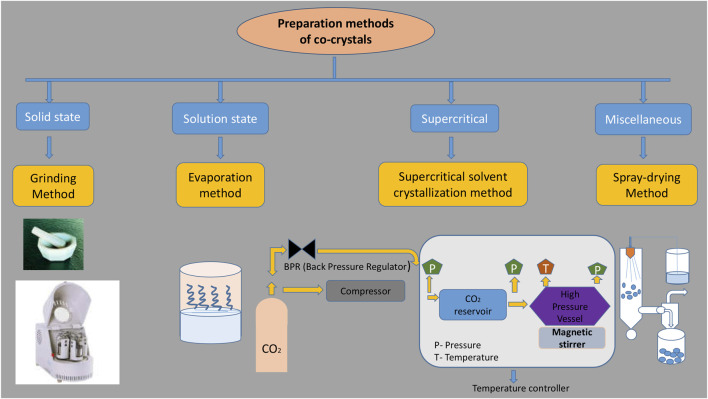
Different types of cocrystallization techniques.

There are various methods available for the preparation of cocrystals, which could be broadly classified into two categories, namely, solid-state based and solution based. Both methods have their own advantages and disadvantages. Solid-state methods are convenient for the preparation of cocrystals on both laboratory and industrial scale, as these techniques can be considered as a convenient, versatile, sustainable, and eco-friendly method for the preparation of cocrystals (Jug and Mura, 2018). Solution-based methods are mainly confined to and convenient for the preparation of cocrystals on a laboratory scale, as they are simple, easy to process, monitor, and control the final product, but at the same time, one should be careful, as solvent selection affects the characteristics of cocrystals (Rodrigues et al., 2018) (Figure 1).

### Solid-State Preparation Methods

#### Contact Formation Method

The contact formation method involves the concept of decreasing the size of the particle by increasing its crystallization rates (Karimi-Jafari et al., 2018). It has also been proven that the crystals that are pre-milled largely contribute toward the spontaneous reaction for the formation of cocrystals (Maheshwari et al., 2009). Many cocrystals have been prepared using this method, proving the fact that the smaller sized particles lead to faster cocrystals formation, for example, urea and 2-methoxybenzamide whose surface energetics increases due to reduction in particle size (Ibrahim et al., 2011.).

#### Solid-State Grinding Method

Solid-state grinding is another method that has been used for many years in the field of research for the preparation of cocrystals. For example, adopting this methodology, liquid-assisted grinding (LAG) in the solid state was used to examine the production of diastereomeric cocrystals of malic and tartaric acids (Eddleston et al., 2012). Primarily, this approach follows two different methods for the preparation of molecular assemblies, such as the neat or dry grinding (DG) method and LAG method.

##### Dry or Neat Grinding Method

In the DG method, the solid form of the API and conformer get ground together manually using a mortar and pestle or mechanically by using a ball mill (Friščič and Jones, 2009; Karimi-Jafari et al., 2018). Brexpiprazole is a drug that lies in BCS Class II. To improve its solubility, the ball milling technique is used and has been found to be one of the convenient methods to prepare cocrystals with conformers, for example, succinic acid and catechol (Boksa et al., 2014). The main problem lies with the dry grinding method, that is, one cannot ensure the formation of a stoichiometry mixing of cocrystals, which requires further an additional step to get a pure cocrystal product.

##### Liquid-Assisted Grinding Method

The LAG method incurs the addition of a small amount of the solvent (to the mixture) in order to get the desired cocrystal product. This added solvent can act as a catalyst for the formation of cocrystals (Karimi-Jafari et al., 2018). Trask et al. (2005) followed the LAG to prepare cocrystals of caffeine and dicarboxylic acid, which indicated that there was an acceleration of reaction kinetics by choosing the suitable solvent for the reaction. Another notable example is the cocrystal of piracetam, a nootropic drug, which was prepared by employing both dry and LAG methods using tartaric acid and citric acid as the conformers. Comparing the above methods, it was observed that the LAG method is the faster method than the dry or neat grinding methods (Rehder et al., 2011). LAG is a more efficient method than the neat grinding method for the screening of cocrystal hydrates. Karki et al. (2007) compared the outcomes of LAG and neat grinding for the screening of cocrystal hydrates of theophylline–citric acid and caffeine–citric acid. They found that LAG gives consistent results irrespective of the reactant's nature (hydrated/anhydrous) compared to neat grinding. Hence, LAG is the preferred method for the screening of pharmaceutical cocrystals. There are several research studies regarding the formation of carbamazepine cocrystals with nicotinamide and saccharin in which the use of the solvent drop method has been proved to be convenient for preparing cocrystals (Weyna et al., 2009).

Despite several advantages of LAG and DG methods such as being inexpensive, easy to perform, and eco-friendly, when it comes to preparing for large-scale production for industries, hot-melt extrusion (HME) is preferred (Gajda et al., 2019).

#### Hot-Melt Extrusion

The HME method is a widely used technique in the pharmaceutical industry. Since the last decade, this method has shown the capability to replace old methods of preparation of cocrystals and be used both in laboratories and commercially (Patil et al., 2016). In this technique, both the API and conformer get mixed simultaneously with the aid of heat and pressure above their melting points (Karimi-Jafari et al., 2018). The HME method for preparing cocrystals was introduced by Medina et al. (2010), where they found that there is an increase in surface contact among the molecules, and homogeneous mixing can significantly assist the production of cocrystals. The proper selection of extruders is highly essential in the case of the HME method. Especially for pharmaceutical cocrystal preparation, a twin-screw extruder could be used to ensure a proper homogeneous mixture of components (Gajda et al., 2019). The temperature is an important factor when the HME method is considered. Dhumal et al. (2010) reported the formation of cocrystal with ibuprofen and nicotinamide; here, they observed that raising the temperature above the eutectic point improves mixing and increases the dissolution rate, and further eliminates the size reduction step. Screw configuration also affects the quality of the cocrystals (Moradiya et al., 2014). Low screw rotational speed is required to get high-quality cocrystals, as high rotational speed may degrade the product (Thiry et al., 2015). HME can be used in a variety of ways; for example, it can act as a reaction vessel to produce cocrystals to enhance the bioavailability of poorly water-soluble APIs. Hence, HME is a reliable method used widely to accept the changing regulatory demands, is solvent free, and is a single-step method that can replace the other old methods.

#### High Shear Wet Granulation

The high shear wet granulation (HSG) method includes assembling the powder components in a liquid medium which could be performed in a high shear granulator (Karimi-Jafari et al., 2018). HSG can also be considered a suitable method for the formation of cocrystal components on the batch scale. Granules formation is quite dependent upon the impeller speed, excipient used, and time of exposure of the granules (Rehder et al., 2013). Proper selection of a liquid media for the granulate in order to get the desired product is absolutely necessary. Veronika et al. successfully prepared cocrystals of ivabradine hydrochloride with the conformer S-mandelic acid *in situ* by the wet granulation method. They studied the influence of excipients on the stability of cocrystals during the wet granulation process and found that excipients do not have an influence on the production of ivabradine–mandelic acid cocrystals (Sládková et al., 2017). The HSG method is convenient for many samples, but it is not appropriate for drugs which are thermally labile and have a complex process. Hence, depending upon the kind of sample and retaining the quality of cocrystal needed to be formed, the desired technique needs to be selected.

### Solution-Based Preparation Methods

The solution-based method is another category of preparation method for cocrystals, which includes evaporative cocrystallization, cooling crystallization, reaction cocrystallization, and isothermal slurry conversion (Karimi-Jafari et al., 2018). The cocrystal operating range is the best tool to create cocrystals from the solution. This range can be found using eutectic points from the solution containing a cocrystal mixture and conformer. This range is explained by the ternary phase diagram (Steed, 2013; Holaň et al., 2014), and it also explains the stability.

#### Evaporative Cocrystallization

In this technique, a solution of cocrystal components (API and conformer) is prepared in a volatile solvent. The solution is kept at room temperature. Due to the slow evaporation of the solvent, the solute components reach their supersaturation concentration which leads to nucleation and crystal growth (Holaň et al., 2014). This technique is suitable for preparing pharmaceutical cocrystals. Guerain et al. (2020) used this method for the preparation of ibuprofen–nicotinamide cocrystals in ethanol solvent. The advantages of this technique are that it is easy to handle, it has high potency while screening, and the process is simple. The limitations of the technique are the excess consumption of precious organic solvents which is harmful to the environment, scale-up is difficult, and there is the possibility of formation of solvates (Malamatari et al., 2017).

#### Cooling Crystallization

In crystallization, temperature plays a key role. For some of the compounds, increasing the temperature leads to an increase in solubility, and for some others like supersaturated solutions, allowing to cool leads to the formation of cocrystals as precipitates. The main lead in this technique is the formation of the most uniform cocrystal size with energy efficiency. Latent heat is taken out by heating the solution, and the remaining are allowed to cool. In this system, the warm solution is circulated and cooled at time intervals using pressure as a function, and it is also combined with the evaporative system in some of the other instruments (Yu et al., 2010; Karimi-Jafari et al., 2018). By using this preparative method, cocrystals of caffeine and glutaric acid in acetonitrile are prepared (Yu et al., 2010). Methods like vacuum cooling crystallizer, continuous cooling crystallizer, and scraped surface cooling crystallizer are the various techniques under this method.

#### Reaction Crystallization

The cocrystals under this method were formed by using a solution containing reactants. Upon addition to another solution and stirred in a vessel, the concentration overcomes the solubility in the mixture that leads to the formation of the crystals. Mostly, in this type of technique, reactions are done very quickly and mixing conditions influence the crystal size (Caro et al., 2014; Karimi-Jafari et al., 2018). Nucleation growth depends on the mixing at the microstate level that gives supersaturation and reduces solubility. It allows nucleation and leads to crystal formation. By using this technique, carbamazepine forms cocrystals with nicotinamide (Rodríguez-Hornedo et al., 2006). This technique can inhibit the formation of a single component crystal, whereas the major drawback of this technique is that it is hazardous to the environment (solvents), solvates in the yields, and is difficult to scale (Malamatari et al., 2017).

#### Isothermal Slurry Conversion

This is the most efficient screening and scale-up method for cocrystallization (Croker and Rasmuson, 2014; Karimi-Jafari et al., 2018). In this method, the conformer and API are dissolved in different solutions at a suitable temperature and allowed to stir for the required time. Then the concentration of constituents exceeds the critical activity of the conformer that allows the nucleation growth that results in the crystal formation (Chadha et al., 2015; Malamatari et al., 2017; Rodrigues et al., 2018). The presence of a stable form of theophylline–aspirin crystal in isopropyl alcohol was observed by isothermal slurry conversion (Darwish et al., 2018). It is an easy way to prepare cocrystals because it involves the use of less apparatus and halts the development of a single component crystal, but its limitations involve being not eco-friendly, as hazardous solvents are employed, and the scale-up being challenging (Rodrigues et al., 2018).

### Supercritical Fluid Methods

Morphology and size reduction can be altered by using this technique (Rodrigues et al., 2018), where we can achieve single-step particle formation. This is the most advantageous technique for cocrystallization where we can get the highest quality of crystals (Velaga et al., 2002; Chadha et al., 2015; Rodrigues et al., 2018). The most commonly used supercritical fluid is CO_2_; the advantages of using this fluid is to reduce the processing steps, being eco-friendly solvent, well-finished products (without solvent), a greater tendency for solubility, and mainly product degradation that is less because of the lower temperatures (31°C, 7.39 MPa) (Ciou and Su, 2016; Douroumis et al., 2017; Sodeifian and Sajadian, 2018).

#### Rapid Expansion of Supercritical Solution

The rapid expansion of the supercritical solution is one of the techniques used to produce fine microparticle crystals (Sodeifian and Sajadian, 2018). The conformer and API solution are depressurized under atmospheric conditions in supercritical fluid CO_2_, then the solvent fluid drops gradually to supersaturation in the supercritical CO_2._ The supersaturation leads to nucleation growth, finally forming the crystals. The solubility of letrozole was improved 7.1 times by this method (Sodeifian and Sajadian, 2018). The drawback of this technique is that only some of the pairs of conformer–drug combinations are soluble in CO_2,_ and it also gives low yields (Douroumis et al., 2017).

#### Supercritical Solvent Crystallization

**FIGURE 2:**
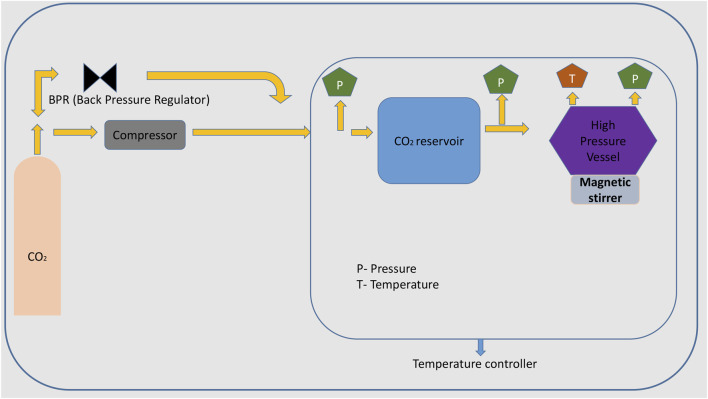
Schematic diagram of supercritical solvent crystallization.

In the supercritical solvent crystallization technique, CO_2_ works as a solvent, so there is no need to add any other organic solvents. In this method, the solvent causes intermolecular interactions that lead to the nucleation growth and formation of crystals. The benefit of this technique is that it can be performed by eliminating the drying steps (Ribas et al., 2019). Solubility can be altered by adjusting the temperature and pressure conditions (of CO_2_). Crystals of carbamazepine with saccharin, theophylline, and indomethacin were obtained with high product yield (Padrela et al., 2015). The advantage of this technique is that it prevents the formation of solvates and hydrates in the crystals because of the absence of water in this method. It is limited to single-component crystal formation (Malamatari et al., 2017) (Figure 2).

#### Supercritical Anti-Solvent Method

**FIGURE 3:**
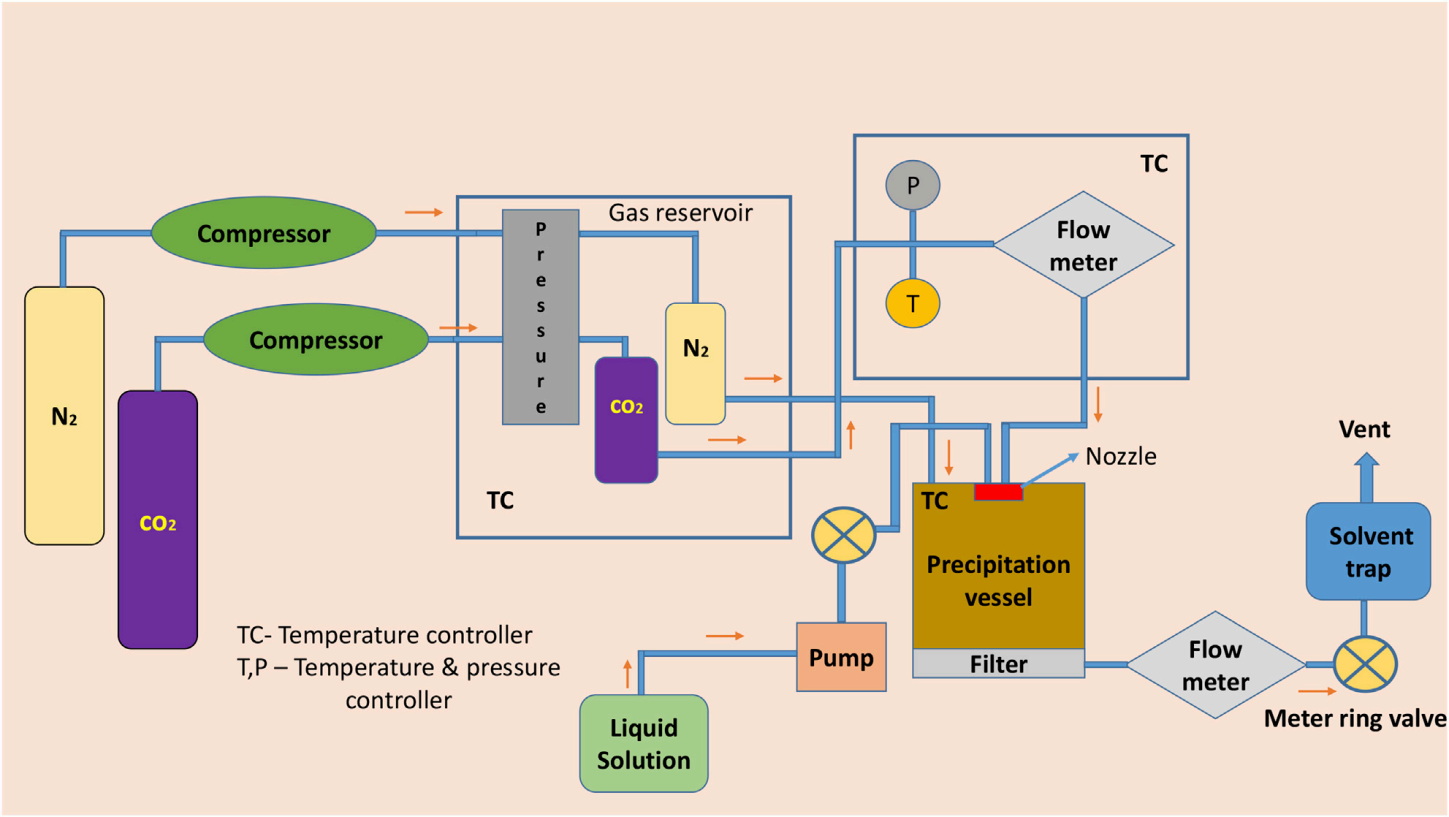
Schematic diagram of supercritical atomization and anti-solvent crystallization and supercritical anti-solvent crystallization.

In the supercritical anti-solvent method, CO_2_ acts as an anti-solvent because of its low solvent power toward the conformer and API, also greater miscibility toward the organic solvents. Ethanol or acetone is a polar organic solvent which dissolves the API and conformer in it. By inducing the solution containing the API and conformer into the high-pressure vessel containing supercritical CO_2_ fluid or using other methods by spraying the solution into the precipitation chamber, the fluid expands the volume and decreases the solubility and leads to supersaturation forms of the cocrystals (Pando et al., 2016; Douroumis et al., 2017; Karimi-Jafari et al., 2018). For example, naproxen–nicotinamide microcrystalline ensemble was prepared by using CO_2_ as an anti-solvent. (Neurohr et al., 2016). It is also a single-step process and the drawback of the technique includes the use of hazardous solvents and many special condition processes are needed for the solvents (Rodrigues et al., 2018) (Figure 3).

#### Supercritical Assisted Spray Drying Method

Other supercritical processes are atomization and supercritical fluid–enhanced atomization (Douroumis et al., 2017), and both these methods work in a similar way. When the solution gets depressurized (cocrystal components along with supercritical CO_2_), the following liquid splits (using coaxial nozzle) into the form of fine droplets, which are sprayed into a drying chamber under atmospheric pressure in the atomization method and into other chambers at required pressures. Crystals need to be collected which are deposited on the walls of the chamber. In both the methods, CO_2_ acts as an anti-solvent, while in the secondary stream, CO_2_ can be replaced by N_2_ gas (Pando et al., 2016; Douroumis et al., 2017; Karimi-Jafari et al., 2018; Rodrigues et al., 2018). Crystals of itraconazole with l-malic acid were prepared by atomization and anti-solvent crystallization methods. In the final crystals, tetrahydrofuran was removed by flushing and dried with more supercritical CO_2_ (Ober et al., 2013)_._ Similarly, supercritical fluid–enhanced crystallization gave the pure form of theophylline crystals by using different conformers (Padrela et al., 2014).

### Miscellaneous Methods

Besides the abovementioned popular methods, there are various methods used for getting the desired cocrystals.

#### Laser Irradiation

The laser irradiation method provides a new way to prepare cocrystals as in this technique, high-power CO_2_ laser is used by varying the raster speed along with the power of the laser which stimulates recrystallization to a cocrystal framework by using the powder form of the conformer. Cocrystals of caffeine with oxalic acid and cocrystals of caffeine with malonic acid were prepared by using this technique (Titapiwatanakun et al., 2016).

#### Electrospray Technology

The electrospray cocrystallization of carbamazepine and itraconazole (Patil et al., 2017), developed with the desired conformer, revealed that the electrospray technique is a unique technique which is a single-step, selective method for the synthesis of cocrystals, which leaves the traditional techniques far behind. This technique involves the occurrence of droplets and charging at the same time by providing an electric field, which leads to the formation of elongated solution droplets.

#### Spray Drying Technique

Spray drying is a unique single-step method that has the capability to transfer solutions, emulsions, or suspensions to solid form (e.g., powder form or agglomerate) (Vehring, 2008). This concept has been used many decades back but has been recently used in pharmaceutical industries as the product at the end ensures proper quality standards in particle size and shape, moisture content of particles, and manages the bulk density (Mudit et al., 2010). Carbamazepine and nicotinamide cocrystals are prepared using the spray drying method more conveniently and a similar quality crystal obtained as formed by the LAG method (Patil et al., 2014). Hence, spray drying is a reliable method for the production of cocrystals on an industrial scale. Besides, this method has bulky and expensive equipment with less thermal efficiency (Mudit et al., 2010).

#### Freeze Drying

The freeze drying method works by freezing and lowering the surrounding pressure, which allows the material to undergo sublimation and hence leads to a phase change (Karimi-Jafari et al., 2018). Freeze drying is a distinctive process that has several advantages over other methods, as it is used in large-scale production, eliminating the issue of differences in solubility of the conformer. Oxalic acid and theophylline (Eddleston et al., 2013) cocrystal preparation concluded that the freeze drying method allows the formation of additional solid forms which cannot be achieved by other standard methods.

#### Electrochemically Induced Cocrystallization

Electrochemically induced cocrystallization can create a potential cocrystallization of ionizable compounds. While preparing a cocrystal of cinnamic acid and 3-nitrobenzamide, the electrochemical application can shift the pH to get neutral carboxylic acids and thus act as a driving tool for the cocrystallization (Urbanus et al., 2011).

#### Resonant Acoustic Mixing

Resonant acoustic mixing includes the mixing of constituents without grinding in the presence of any suitable liquid to get the desired cocrystal. Several cocrystals of carbamazepine (Am Ende et al., 2014) have been formed using this method, with the addition of different solvents. Indeed the benefit of this process is that it can easily produce cocrystals on a large scale since it preblends and then cocrystallizes. Furthermore, it could re-slurry in the same equipment, which is a boon for the pharma industries. With the ever-growing demand for cocrystals in the market, the demand for the proper equipment and the knowledge of the desired method, both are required. The above study aimed at highlighting the different methods of getting a suitable cocrystal, which could further be characterized using various techniques.

## Characterization Methods of Cocrystal

Characterization techniques are essential while carrying out research work on cocrystals. There are various characterization methods to determine the structure and properties of pharmaceutical cocrystals. X-ray diffraction (XRD) techniques [both powder XRD (PXRD) and single-crystal XRD (SCXRD)] are crucial techniques to determine the structure of cocrystals as these resolve bond angles, bond strengths, and torsion angles of molecules using an X-ray beam. Both PXRD and SCXRD techniques can be used depending upon the type of the samples, whether they are in the crystalline or powder form. The PXRD technique is used to observe the phase transformation properties of some of the cocrystals (Chappa et al., 2018). The SCXRD technique can be useful in predicting the intermolecular interactions (hydrogen bond interactions) between the molecules, as these give an idea about the overall geometry of the molecules (Luo and Sun, 2013). The XRD technique can sometimes be challenging as it involves long-range ordering, unlike the case of solid-state nuclear magnetic resonance (SSNMR), which is specific to local orderings (Deng et al., 2007). SSNMR is a useful tool for those kinds of research where a suitable single crystal cannot be found to examine using the XRD technique (Chen, 2014). The presence of low-resolution spectra in SSNMR can be a hindrance in determining the structure of the sample, but the introduction of the magic-angle spinning technique can lead to the production of better resolution spectra (Polenova et al., 2015). SSNMR spectroscopy is useful in obtaining high-resolution data about the structure if the solid phase cannot be characterized using SCXRD. Fourier-transform infrared (FTIR) is a reliable technique to find the interaction between the API and conformer. It plays an important role in detecting the formation of hydrogen bonds with the conformer (Lin et al., 2014). For example, `O-H…O hydrogen bonding between -OH and -C=O functionalities can be justified by a shift in the stretching frequency of O-H and C=O (Bhandaru et al., 2015). Besides determining the structure of the sample, having an idea about the physical properties of the newly formed sample is also necessary, which can be observed using thermoanalytical techniques, like differential scanning calorimetry (DSC) and thermogravimetric analysis, which are characterization techniques to determine the thermal properties of cocrystals. DSC thermograms play an important role in showing the melting endotherm maximum of a sample. This information about melting endotherms maxima may give an idea about a new complex phase (Elbagerma et al., 2010). DSC also presents the novel behavior of a crystallized mixture during heating and cooling. The change in heat of fusion and heat of crystallization with a change in concentration can be observed during the DSC technique (Miller and Patel, 1981). Thermogravimetric analysis is one of the characterization techniques of crystal engineering. It is used to find the loss of mass when the sample is heated to certain temperatures with respect to the time (Qiao et al., 2017). This technique helps to find out physical activities (Dai et al., 2020) like sorption, absorption, and desorption and chemical activities like chemisorption, thermal decomposition, oxidation, and reduction. It is mainly used to determine the thermal stability of the sample (Wang et al., 2020) in an artificial atmosphere like a vacuum, inert gas purging, where the control pressure can also be created based on the requirement of the sample (Horstman et al., 2015). Differential thermal analysis (DTA) is one of the thermoanalytical techniques, which is somehow related to the DSC technique. The only existing difference is that DSC can measure the heat flow of samples with varying temperatures, but with DTA, we can measure the heat difference between the sample of interest and the reference sample with varying temperatures. DSC and TGA are reliable techniques to determine thermal properties, but cocrystal screening to determine properties such as solid-state transition, miscibility, crystalline nature, and morphology can be better studied using hot stage microscopy (HSM) (Malamatari et al., 2017). HSM is one of the widely used techniques for cocrystal, as it is relatively faster as it eliminates the need to prepare cocrystal from conventional methods (Kumar et al., 2020). Besides FTIR, Raman spectroscopy is known to be one of the vibrational spectroscopic techniques as it can be used to predict various bands, whether there is the formation of a new crystal which can be observed by the change in the vibrational frequency of the obtained sample (Elbagerma et al., 2010). Although, FTIR is a favorable technique, the signal-to-noise ratio is higher in the case of time-dependent terahertz (THz) domain spectroscopy when compared with far FTIR (Han et al., 2001). THz time-domain spectroscopy uses frequency ranges between microwaves and near-infrared (NIR) and is hence considered as one of the vibrational spectroscopic techniques (Walther et al., 2010) and is useful in that by examining the absorption spectra, it is possible to determine if a cocrystal is developed or not. Determining the crystal morphology using the electron diffraction technique is preferably easy when compared with the XRD technique (Zhou and Greer, 2016). Although XRD technique data are more accurate, electron microscopy technique is known to complement in determining the structure (McCusker and Baerlocher, 2009). SEM technique can be used to compare the morphology of the cocrystals formed with the morphology of the starting material and can indicate about the formation of new phases (Liu et al., 2016). SEM can detect the properties of a given sample such as surface topography, composition, and crystal orientation in the nanoscale range. It can also provide information about crystal defects (if any) by modifying different intensities of the incident beam of radiation (Naresh-Kumar et al., 2020).

X-ray photoelectron spectroscopy (XPS) is another important technique for determining the nature of molecular solids (cocrystal/salt) by studying changes in the binding energy of the core electrons on the atoms which participate in hydrogen bonding or proton transfer. XPS gives information about the extent of proton transfer in acid–base molecular crystals. It is used to explain whether the formed product is a salt or cocrystal. In a salt, there is transfer of protons which leads to more shift in the binding energy of core electrons of the atoms participating in proton transfer, but in a cocrystal, there is no proton transfer between the molecules, so there will be very less shift in the binding energy of the core electrons. Schroeder et al. showed for the first time that it is possible to differentiate between a salt and cocrystal by observing the shifts in the binding energies of the core electrons (Stevens et al., 2015). Srinu et al. studied the possibility of differentiating salt–cocrystal continuum from salt and cocrystal by using XPS. They found that it is not possible to differentiate between the salt–cocrystal continuum from salt and cocrystal because of the lack of clear distinction between intermediate binding energies and salt–cocrystal binding energy values (Tothadi et al., 2021). Hence, it can be concluded that every technique has some characteristic features which are different from others, and to characterize a cocrystal sample, every technique can bring conclusive information about cocrystals.

## Application of Cocrystallization Techniques in the Pharmaceutical Industry

To harness the ability of cocrystals for the production of improved drug forms, we need to optimize/develop cocrystallization techniques for industrial purposes. Industrial production of cocrystals requires scalable, robust, and environmentally friendly cocrystallization techniques. The quality of the product should not be compromised by large-scale production. API-conformer lability, solubility and stability of components, vulnerability to form polymorphs, amorphous states or solvates of components are the criteria for the selection of the cocrystallization technique. The purity, morphology, and particle size distribution of cocrystals are greatly influenced by the choice of the cocrystallization technique (Rodrigues et al., 2018). Despite a large number of reported cocrystallization techniques, very few methods are scalable. Spray drying, spray congealing, and HME are some of the scalable cocrystallization techniques (Douroumis et al., 2017). Fruitful application of these cocrystallization techniques in industrial setup requires an in-depth understanding of the theory of the technique, process parameters that need to be controlled to get a high yield and good quality product, and the effect of other excipients on the cocrystal composition during manufacturing.

Spray congealing is a relatively new cocrystallization technique in which molten API and conformer mixture is passed through an atomizer. The atomizer breaks the liquid into fine droplets, and these droplets are cooled by a cocurrent stream of cooling gas. This process leads to the solidification of liquid into fine particles, forming cocrystals. Since the lack of usage of any solvents, spray congealing is a green technique (Duarte et al., 2016). It permits tuning of the particle properties of cocrystals, such as particle size, shape, purity, and flow properties, by changing process parameters like atomization and cooling efficiency (Duarte et al., 2016). It is considered a hybrid technique between spray drying and HME. It is a scalable, cost-effective, and green technique. The disadvantage of the spray congealing technique is the requirement to melt the components, which limits its usage for thermolabile APIs. Duarte et al. (2016) showed that spray congealing could be used in the production of cocrystals. They successfully produced caffeine:salicylic acid and carbamazepine:nicotinamide cocrystals by using spray congealing, and they also demonstrated that the particle properties of caffeine:glutaric acid cocrystals can be tuned by adjusting the process parameters like atomization and cooling-related parameters.

Spray drying is a solvent-based cocrystallization technique. Urano et al. (2020) prepared cocrystals of cilostazol with three conformers (4-hydroxybenzoic acid, 2,4-dihydroxybenzoic acid, and 2,5-dihydroxybenzoic acid) using the spray drying technique. They compared the cilostazol cocrystals prepared from different cocrystallization techniques and found that the cocrystals produced by spray drying were the same as those prepared from other techniques. Furthermore, they found that the dissolution behavior of cilostazol cocrystal made from spray drying was improved compared to those made from other techniques. Spray drying is a scalable cocrystallization technique. Walsh et al. (2018) compared the efficiency of spray drying and HME processes in producing cocrystals of ibuprofen and isonicotinamide in the presence of excipients and concluded that spray drying yielded good quality cocrystals in the presence of excipients. Patil et al. (2014) successfully developed carbamazepine:nicotinamide cocrystals by spray drying, and they compared these cocrystals with those produced by LAG. They found that both cocrystals exhibit the same quality. This indicated that spray drying can be used to produce pharmaceutical cocrystals. Since spray drying is simple, scalable, and cost-effective, it can be utilized for industrial manufacturing of pharmaceutical cocrystals. This method needs high amounts of hazardous organic solvents, so it is not a green technology.

HME is a solvent-free cocrystallization method. Jafari et al. studied HME as a manufacturing technique for a model cocrystal system ibuprofen:nicotinamide. They succeeded in achieving the total conversion of constituents into a cocrystal product by optimizing the process parameters like screw speed and temperature. They also studied the effect of polymeric excipient (Soluplus) on the cocrystallization temperature and found that the cocrystallization temperature was reduced by the presence of the polymeric excipient, and the mechanical properties of the cocrystals also improved (Karimi-Jafari et al., 2019).

Boksa et al. (2014) successfully produced the cocrystals of carbamazepine:nicotinamide in the presence of a polymer matrix (Soluplus) by HME. This matrix-assisted cocrystallization process produced high-quality cocrystals with an 80:20 (w/w) cocrystal:matrix ratio. These cocrystals showed improved dissolution and solubility properties than the original forms. This indicates that the matrix also influences the solubility and dissolution properties of cocrystals. This study shows that the HME technique can be applied for the industrial production of pharmaceutical cocrystals because it is scalable and facilitates continuous manufacturing by simultaneously mixing the excipients to the feed of API and conformer (Boksa et al., 2014). Yu et al. reported that an increase in the cocrystal yield (ibuprofen:isonicotinamide in xylitol matrix) can be achieved by choosing the optimum temperature and screw configuration. The optimum temperature was selected from phase diagrams, which were constructed from Flory–Huggins solution theory using melting point depression measurements. It was shown that incorporating screws that facilitate intensive mixing/kneading also improves the cocrystal yield (Li et al., 2018). Butreddy et al. (2020) prepared aripiprazole–adipic acid cocrystals in the presence of a 5% Soluplus polymer matrix using HME. The cocrystals produced showed improved solubility and dissolution properties. Kelly et al. (2012) showed that the NIR spectroscopy probe can be inserted into the twin-screw extrusion technique to monitor the cocrystal formation (ibuprofen:nicotinamide) in real time. They reported that NIR can be used as a process analytical technology (PAT) tool to monitor the cocrystallization process in solvent-free extrusion technique.

### Quality-By-Design and Process Analytical Technology

A recent approach to the quality control of pharmaceutical products is quality by design (QbD), proposed by the International Conference on Harmonization (ICH). ICH defined QbD as “a systematic approach to development that begins with predefined objectives and emphasizes product and process understanding and process control, based on sound science and quality risk management.” The aim of the QbD approach is to produce high-quality pharmaceutical products by understanding product and process and implementing this knowledge in designing the production process. PAT is compatible with the QbD approach (Rodrigues et al., 2018). The FDA defined PAT as “a system for the design, analysis, and control of manufacturing processes through timely measurements of critical quality and performance attributes of raw and in-process materials and processes, with the goal of ensuring final product quality.” PAT tools play a very important role in designing controlled and optimized processes by providing important physical, chemical, and biological characteristics of the process. This leads to scientific, risk-managed pharmaceutical development, manufacture, and quality assurance (Rodrigues et al., 2018; Panzade et al., 2020).

Continuous manufacturing is a novel process, which was developed as an alternative to the batch process. The batch process is a discontinuous method of manufacturing, whereas continuous manufacturing involves a single continuous process of manufacturing to final products. By implementing a continuous process of manufacturing, we can reduce material waste, energy consumption, and scale-up problems. This method is easy to scale up and facilitates complete automation of product manufacturing. From the above literature examples, HME seems to be the suitable cocrystallization technique in terms of scalability, process control parameters, PAT tools, and continuous manufacturing. But it is not suitable for thermolabile drug molecules (Rodrigues et al., 2018; Panzade et al., 2020).

## Regulatory Guidelines for Pharmaceutical Cocrystals and Their Scale Up

Complying with the regulatory guidelines is important for the successful development of a commercial formulation. USFDA and European Medicines Agency (EMA) issued guidelines for pharmaceutical industries for pharmaceutical cocrystals. USFDA considers pharmaceutical cocrystals as novel crystalline solid forms that enable the enhancement of stability, bioavailability, and processability properties of APIs. Pharmaceutical cocrystals are clearly distinguished from salts by stating that the components present in the crystalline lattice of cocrystals should interact nonionically. Cocrystals are closely related to solvates except that the conformer is not a liquid when it is in pure form at ambient conditions. For the cocrystal-based drug to get approval from the FDA as an new drug application (NDA) or as an abbreviated NDA (ANDA), the applicant has to prove that the interaction between API and conformer is nonionic with the help of the ∆pKa rule, which states that for the formation of cocrystal, difference between pKa values of cocrystal components should be less than 0 or by any analytical evidence and has to prove that the API and conformer dissociate before reaching the site of action for pharmacological activity by *in vitro* dissolution and/or solubility studies. From the regulatory perspective, cocrystals of an API are considered as different polymorphs of the same API but not considered as a new API. Drug–drug cocrystals with or without inactive conformer are considered as fixed-dose combination products and not as a new single API (Center of Drug Evaluation and Research, 2018).

EMA considers cocrystals as a subgroup of solvates. Cocrystals of an API are not considered as a new active form because when administered orally, the cocrystals will dissociate and expose the same API to the site of action. New active form status can only be granted to a cocrystal when the efficacy and safety of the cocrystal are significantly enhanced compared to the pure API. Cocrystals are permitted for generic application if they exhibit similar safety and/or efficacy. Safety and quality of the conformer are also very important for regulatory approval if it was not used in medicinal products previously. Drug–drug cocrystals will be considered as another fixed-dose combination but not as a new active compound unless the cocrystal exposes a new active moiety to the site of action (Madicines Agency, 2015).

## Commercially Available Cocrystals

**FIGURE 4:**
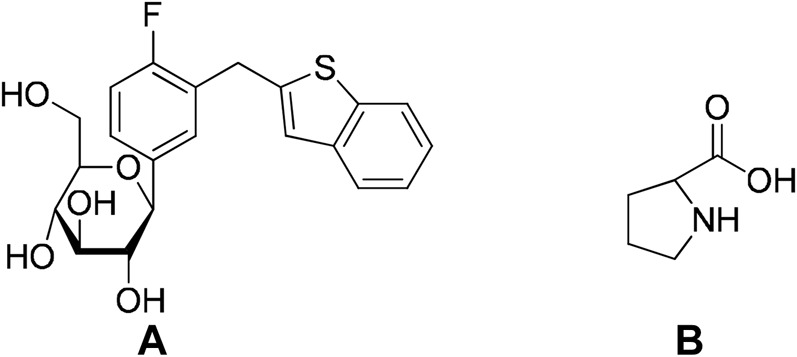
Molecular structures of A. ipragliflozin (API) and B. l-proline (conformer).

Successful application of cocrystallization to the pharmaceutical industry is evident from the approval of drugs that are based on cocrystals in the market. Suglat^®^, Entresto^®^, and Steglatro^®^ are the marketed drugs that contain cocrystal-based APIs (Kavanagh et al., 2019) (Table 1). Suglat^®^ is used in the treatment of diabetes, where ipragliflozin is the API and l-proline is the conformer. Ipragliflozin is a sodium–glucose cotransporter 2 inhibitor which suffers from nonstoichiometric absorption of water (conversion to hydrate) under storage conditions. Cocrystallization with l-Proline imparts stability against hydrate formation. Suglat^®^ was developed by Astellas Pharma and Kotobuki Pharmaceutical and got approval to market in Japan in 2014 (Kavanagh et al., 2019) (Figure 4).

**TABLE 1:** Commercially available pharmaceutical cocrystals.

Commercial name	API	Conformer	Improved property	References
Suglat^®^	Ipragliflozin	l-Prolin	Stability against hydrate formation	(Approval of Suglat tablets, kotobuki Pharmaceuticals, 2014; Kavanagh et al., 2019)
Entresto^®^ (Drug–Drug cocrystal)	Valsartan	Sacubitril	Improved pharmacokinetics and bioavailability of valsartan	(Emami et al., 2018; “; Entresto, Highlights of prescribing information, 2015; Feng et al., 2012)
Steglatro^®^	Ertugliflozin	Z-Pyroglutamic acid	Improved stability	(Duggirala et al., 2019; Vasoya et al., 2019)
Depakote^®^	Valproic acid	Valproate sodium	Solid phase stability and less hygroscopicity	(Brittain, 2013; Kavanagh et al., 2019)
Lexapro^®^	Escitalopram	Oxalate	Improved stability of API	(Lexapro, 2002; Harrison et al., 2007)
Beta chlor^®^	Chloral hydrate	Betaine	Improved thermal stability	(O’Nolan et al., 2016; Kavanagh et al., 2019)

**FIGURE 5:**
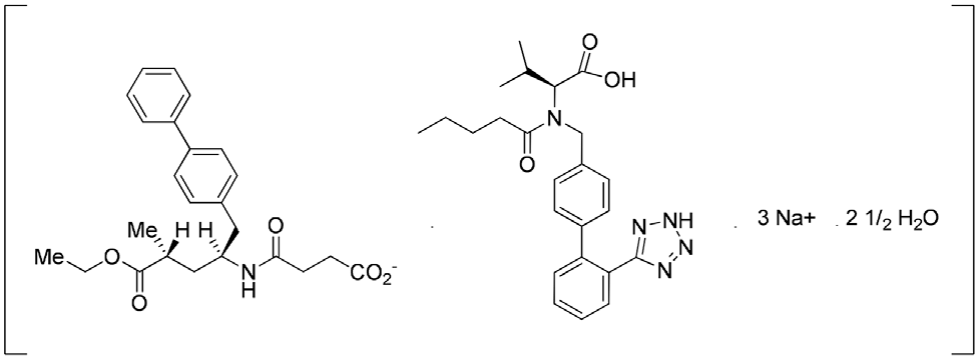
Molecular complex of valsartan and sacubitril (multidrug cocrystal).

Entresto^®^ is a drug–drug cocrystal developed by Novartis. It is a medication for reducing the risk of heart failure. It is a fixed-dose combination of valsartan and sacubitril. Valsartan is an angiotensin II receptor blocker and sacubitril is a neprilysin inhibitor. Entresto^®^ includes a complex of anionic forms of valsartan, sacubitril, sodium cations, and water molecules in the molar ratio of 1:1:3:2.5, respectively, and other excipients. The crystal structure of Entresto^®^ was studied by Feng et al. (2012). This drug is a good example of the improvement of the pharmacokinetics of active ingredients due to cocrystallization. The bioavailability of valsartan in Entresto^®^ is 50% more than the valsartan administered alone (Entresto, 2015.; Kavanagh et al., 2019) (Figure 5).

**FIGURE 6:**
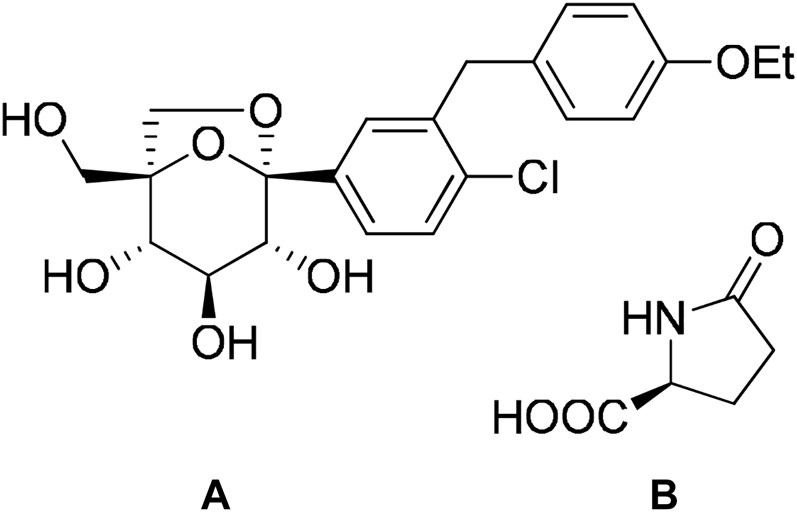
Chemical structures of A. Ertugliflozin and B. l-pyroglutamic acid.

Steglatro^®^ is a medication for type-2 diabetes mellitus. It contains ertugliflozin, a sodium–glucose cotransporter 2 inhibitor, and l-pyroglutamic acid as a conformer. This is an example of improvement of stability of active ingredients by cocrystallization. Ertugliflozin is an unstable amorphous material. The stability and physicochemical properties of ertugliflozin are improved by cocrystal formation with l-pyroglutamic acid in a 1:1 ratio (Kavanagh et al., 2019; staglatro, 2017) (Figure 6).

**FIGURE 7:**
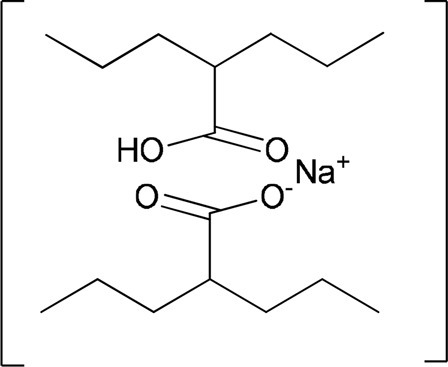
Molecular structure of Depakote^®^

For some drugs, cocrystal forms were developed at a later stage, and for some others, they were identified as cocrystals after some years of approval (Kavanagh et al., 2019). Valproic acid is an approved medication for epilepsy. It exists as an acid form and a sodium salt (sodium valproate) form. The acid form is liquid at ambient conditions and sodium salt is highly hygroscopic. The cocrystal form contains both valproic acid and sodium valproate in a 1:1 ratio (Kavanagh et al., 2019). This cocrystal form is less hygroscopic than the components. Commercially, it is called by different names, such as Depakote^®^, Epilim, and divalproex sodium. The crystal structure of valproic acid and valproate is sustained by a sodium oxygen cluster surrounded by the lipophilic tail of valproate. This structure is considered salt–cocrystal (Brittain, 2013) (Figure 7).

**FIGURE 8:**
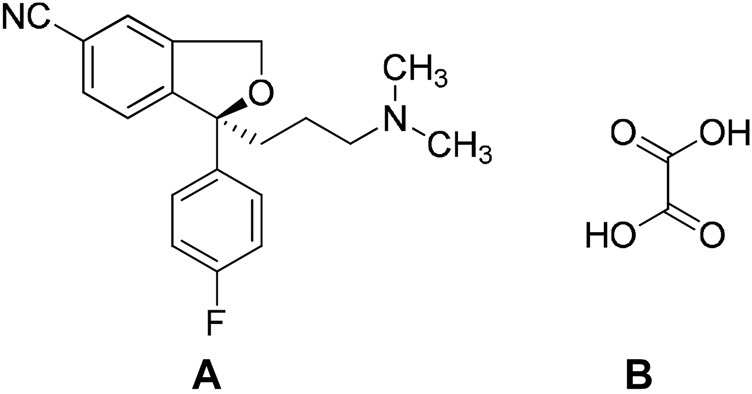
Molecular structures of A. escitalopram and B. oxalic acid.

Escitalopram oxalate (Lexapro^®^) is another example of a drug which has been identified as a cocrystal at a later stage (Kavanagh et al., 2019). It is a selective serotonin reuptake inhibitor which is indicated as a medication for depression. Escitalopram is a pure S-enantiomer of racemic citalopram which is also an antidepressant medication (Lexapro, 2002). It was found that the cocrystal of escitalopram oxalate contains escitalopram cation forming a salt by way of two hydrogen bonds to the same oxalate dianion (N^+^-H^…^O^−^, O^−^); this unit is linked into chains by a neutral oxalic acid molecule (Harrison et al., 2007) (Figure 8).

**FIGURE 9:**
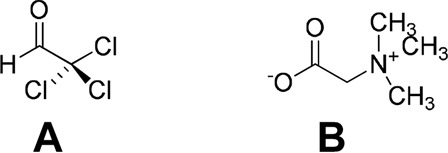
Molecular structures of chloral and betaine.

Chloral betaine (beta-chlor^®^) is another example, which was identified as a cocrystal at a later stage (2016) (Kavanagh et al., 2019). Chloral is a sedative drug. The components of the cocrystal are chloral hydrate and betaine. The formation of cocrystal imparts thermal stability to the parent compound. The melting point of the cocrystal was reported to be 120^°^C, whereas the melting point of chloral hydrate is 60^°^C. In the cocrystal, chloral betaine exists as a charge-assisted diol-carboxylate heterodimer, with further Cl^….^O interactions forming a tetramer (O’Nolan et al., 2016; Kavanagh et al., 2019) (Figure 9).

Generic drugs have a huge market share in the pharmaceutical industry. Cocrystals can contribute to the creation of generic drugs which show similar properties with improved stability compared to the original drug. Recently, Teva api company synthesized a cocrystal of ibrutinib (an anticancer drug prescribed for chronic lymphocytic leukemia) with fumaric acid, which exhibits similar solubility as the original drug but with improved stability. FDA approval is pending for this cocrystal (Teva api, 2020).

## Challenges Associated in the Development of Cocrystals

The primary challenge in the development of pharmaceutical cocrystals is the selection of suitable conformers. Theoretically, the number of possible conformers is very high, so there is a need to develop a screening tool that can predict the probable conformers. Thereafter, these predicted conformers should be experimentally screened to check for the formation of cocrystals (Jones et al., 2006). Due to the intensified research in the field of cocrystals during the past decades, sufficient data have been accumulated to predict the probable conformers. Hydrogen bond propensity, Cambridge Structural Database, supramolecular synthon approach, pKa rule, and Hansen solubility parameter are some of the successful approaches for screening of conformers, but the development of more effective screening tools is necessary for the successful application of cocrystallization to the pharmaceutical industry (Kumar and Nanda, 2018).

For screening cocrystals, different cocrystallization methods can be used. Solvent-based cocrystallization methods present different challenges like selecting a suitable solvent, variations in the solubility of API and conformer in the given solvent (congruent and incongruent), concentration effects, choosing correct heating and cooling profiles, etc. The solid-state grinding method is better than solvent-based methods for the screening of cocrystals. But sometimes solid-state grinding induces phase transformations in pharmaceutical cocrystals (Chow et al., 2017; Kaur et al., 2019). All the selected conformers may not produce cocrystals with desirable physicochemical, pharmacokinetic, and processability properties (Schultheiss and Newman, 2009). The challenges involved in the synthesis and characterization of cocrystals include the formation of salts or solvates or hybrids, inherent instability of cocrystals, instability of cocrystals in solution phase, i.e., variation in the stability, dissolution profile, solubility of cocrystals based on pH, ion concentration, surfactant concentration (Lange et al., 2016; Ren et al., 2019), conversion to the less soluble parent drug form in solution, and polymorphism of cocrystals (Porter et al., 2008; Aitipamula et al., 2010; Kale et al., 2017; Guerain et al., 2020). Thus, the challenges that need to be addressed during cocrystal preparation are the probability of dissociation of cocrystal in the formulation due to interaction with formulation components (excipients), replacement of conformers by excipients, change in the stoichiometry of cocrystal, and conversion to a less soluble parent drug during dissolution (Qiu and Li, 2015; Duggirala et al., 2017). There are no well-established scale-up techniques for the production of pharmaceutical cocrystals. HME, spray drying, spray congealing, and supercritical fluid technology are currently in use for the scale-up of cocrystals. Another challenge in the scale-up of cocrystals is reproducible control of stoichiometry (Karimi-Jafari et al., 2019), a lack of *in vitro–in vivo* correlation properties of cocrystals, which can reduce the development time significantly (González-García et al., 2015; Yousef and Vangala, 2019). The availability of few cocrystal-based drugs in the market indicates that the challenges associated with the development of cocrystals can be conquered.

## Concluding Remarks and Future Perspectives

From the above discussion, we can conclude that the phenomenon of cocrystallization is a better alternative than conventional methods like salt formation, solvates, and polymorphs, to improve the physicochemical and processability characteristics of APIs. An inherently limited number of solvates, polymorphs, and lack of suitable ionizable groups in some APIs limit the application of salts, solvates, and polymorphs in the development of APIs into pharmaceutically acceptable forms. The research during the last decade contributed to the evolution of various aspects of cocrystallization, such as cocrystal screening and development, characterization, production methods, and formulations. Guidelines from regulatory authorities (USFDA, EMA) regarding pharmaceutical cocrystals testify the established role of pharmaceutical cocrystals in the development of better candidates with improved characteristics. Still, this method has not become a routine method for the production of pharmaceuticals. For the standard application of cocrystals in the pharmaceutical industry, research needs to be focused on standardizing synthetic procedures of cocrystals, identifying and optimizing the parameters that influence the quality of cocrystals (purity, yield, and reproducibility), and cocrystal screening and development should be included in the drug development process.
